# Crystal structure of 3-(4-hy­droxy­phen­yl)-2-[(*E*)-2-phenyl­ethen­yl]quinazolin-4(3*H*)-one

**DOI:** 10.1107/S2056989016004473

**Published:** 2016-03-22

**Authors:** Inese Mierina, Dmitrijs Stepanovs, Jolita Kuginyte, Artur Janichev, Mara Jure

**Affiliations:** a Institute of Technology of Organic Chemistry, Faculty of Materials Science and Applied Chemistry, Riga Technical University, Str. P. Valdena 3/7, Riga, LV 1048, Latvia; b Latvian Institute of Organic Synthesis, Str. Aizkraukles 21, Riga, LV 1006, Latvia; c Institute of Synthetic Chemistry, Kaunas University of Technology, Str. K. Barsausko 59, Kaunas, LT 51423, Lithuania

**Keywords:** crystal structure, 2,3-disubstituted quinazolin-4(3*H*)-one, styrylquinazolinone conjugation system, hydrogen bonding

## Abstract

The title compound, C_22_H_16_N_2_O_2_ {systematic name: 3-(4-hy­droxy­phen­yl)-2-[(*E*)-2-phenyl­ethen­yl]quinazolin-4(3*H*)-one}, consists of a substituted 2-[(*E*)-2-aryl­ethen­yl]-3-aryl­quinazolin-4(3*H*)-one skeleton. The substituents at the ethyl­ene fragment are located in *trans* positions. The phenyl ring is inclined to the quinazolone ring by 26.44 (19)°, while the 4-hy­droxy­phenyl ring is inclined to the quinazolone ring by 81.25 (8)°. The phenyl ring and the 4-hy­droxy­phenyl ring are inclined to one another by 78.28 (2)°. In the crystal, mol­ecules are connected *via* O—H⋯O hydrogen bonds, forming a helix along the *a*-axis direction. The helices are linked by C—H⋯π inter­actions, forming slabs parallel to (001).

## Abstract 2

The title compound consists of a substituted 2-[(*E*)-2-aryl­ethen­yl]-3-aryl­quinazolin-4(3*H*)-one skeleton. The substituents at the ethyl­ene fragment are located in *trans* positions. In the crystal, mol­ecules are connected *via* O—H⋯O hydrogen bonds forming a 2_1_ helix propagating along the *a*-axis direction.

## Chemical context   

Compounds containing the 2-[(*E*)-2-aryl­ethen­yl]-3-aryl­quinazolin-4(3*H*)-one core are well known for their broad biological activities. These compounds demonstrate anti­biotic effect *in vivo* against methicillin-resistant *Staphylococcus aureus* (Bouley *et al.*, 2015 ▸; Chang *et al.*, 2014 ▸) and anti­leishmanial activity (Birhan *et al.*, 2014 ▸). 2-Styryl functional­ized quinazolinones are applicable as anti­cancer agents against human cell lines (Kamal *et al.*, 2013 ▸; 2012 ▸; 2010**a* ▸,b* ▸) and anti­convulsants (Das *et al.*, 2014 ▸). Analogues of the title compound are Hsp90 inhibitors with *in vitro* anti-tumor activity (Park *et al.*, 2007 ▸), as well as suppressants of the ubiquitin ligase activity of a human polypeptide (Erez & Nakache, 2011 ▸), GluN2D-containing NMDA receptors (Hansen & Traynelis, 2011 ▸) and c-KIT expression (Wang *et al.*, 2013 ▸). Compounds with such a structure are good modulators of both γ-secretase (Fischer *et al.*, 2011 ▸) and Rho C activity (Sun *et al.*, 2003 ▸), as well as AMPA receptor antagonists (Chenard *et al.*, 2001 ▸; 1999 ▸; Welch & DeVries, 1998 ▸). Piriqualone (the 2-hetaryl­vinyl analogue of the above mentioned compounds) has been used as a sedative–hypnotic drug (Kumar *et al.*, 2015 ▸).
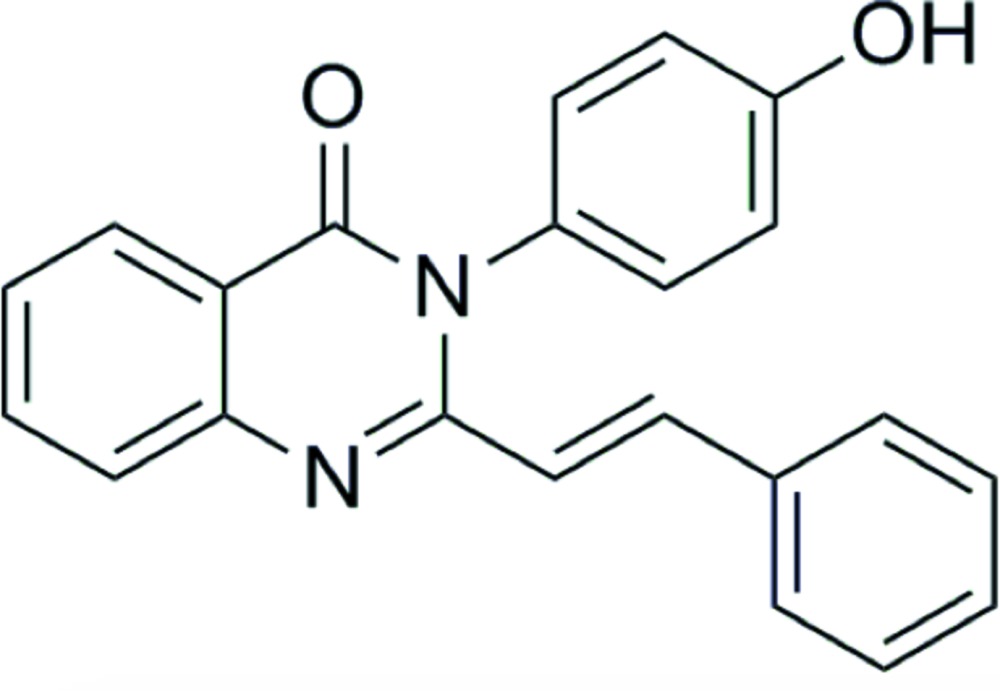


## Structural commentary   

**Figure 1:**
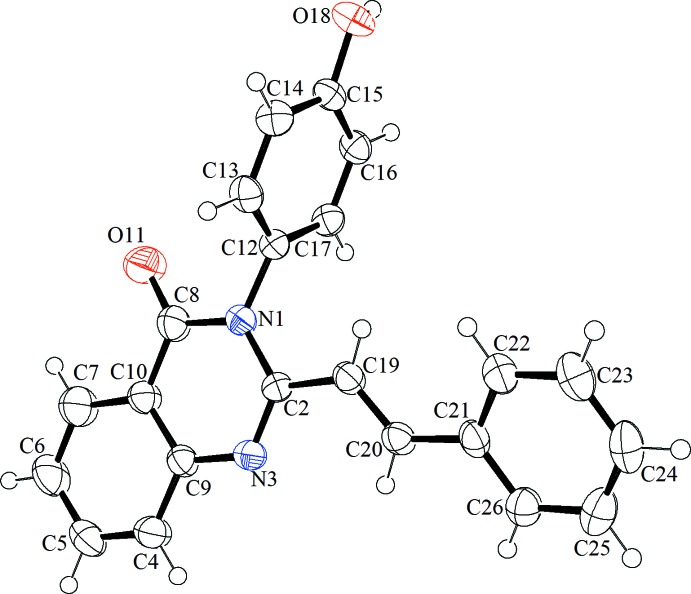
The mol­ecular structure of compound **1**, showing the atom labelling. Displacement ellipsoids are drawn at the 50% probability level.

The title compound **1**, Fig. 1 ▸, consists of a substituted 2-[(*E*)-2-aryl­ethen­yl]-3-aryl­quinazolin-4(3*H*)-one skeleton. The substituents at the ethyl­ene fragment are located in *trans*-positions. Unlike the structure reported by Nosova *et al.* (2012 ▸), where the conjugation system of styrylquinazolinone is practically planar, in compound **1** the 2-phenyl­eth-(*E*)-enyl substituent is twisted with respect to the plane of the quinazolone ring. The phenyl (C21–C26) and the 4-hy­droxy­phenyl (C12–C17) rings are inclined to one another by 78.2 (2)°, and to the quinazolone ring (N1/N2/C2/C4–C10) by 26.44 (19) and 81.25 (8)°, respectively. A similar styryl­quinazolinone conjugation system geometry has been found in structures reported previously (Trashakhova *et al.*, 2011 ▸; Ovchinnikova *et al.*, 2014 ▸).

## Supra­molecular features   

**Figure 2:**
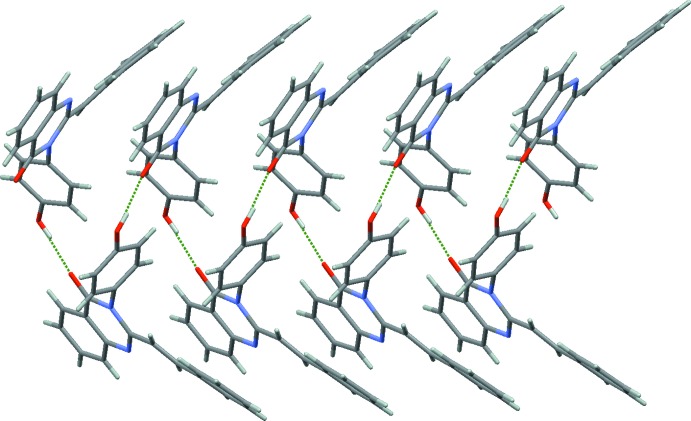
A fragment of the crystal structure of compound **1**, showing the helix-like hydrogen-bonded chain propagating along the *a*-axis direction.

**Figure 3:**
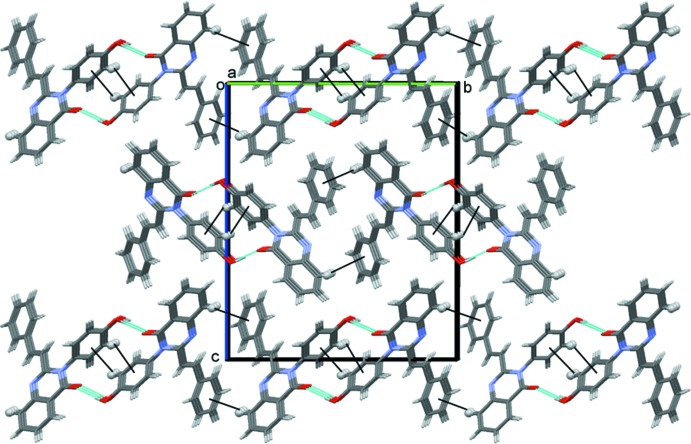
A view along the *a* axis of the crystal packing of compound **1**. The hydrogen bonds are shown as dashed lines and the C—H⋯π inter­actions (see Table 1 ▸) are represented as thin black lines.

In the crystal of **1**, mol­ecules are connected *via* O—H⋯O hydrogen bonds forming a 2_1_ helix, with graph set *C*(3), propagating along the *a-*axis direction (Table 1 ▸ and Fig. 2 ▸). This is similar to the crystal packing reported for the structure of diltiazem acetyl­salicilate hydrate (Stepanovs *et al.*, 2016 ▸). In **1**, the helices are linked *via* C—H⋯π inter­actions, forming slabs lying parallel to the *ab* plane (Table 1 ▸ and Fig. 3 ▸).

## Database survey   

**Figure 4:**
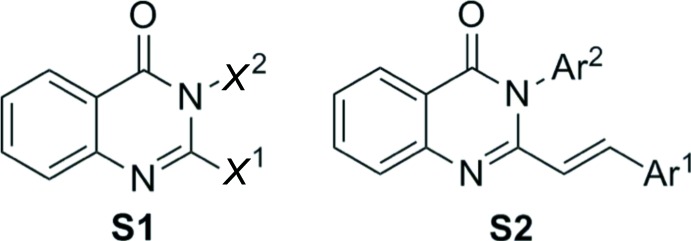
Substructures used for the Database survey.

A search of the Cambridge Structural Database (Version 5.37; Groom & Allen, 2014 ▸) for substructure **S1** (Fig. 4 ▸) gave 137 hits, while a search for substructure **S2** (2-aryl­vinyl 3-aryl quinazolin-4(3*H*)-one skeleton, Fig. 4 ▸) gave only three hits: Nosova *et al.* (2012 ▸); Trashakhova *et al.* (2011 ▸); Ovchinnikova *et al.* (2014 ▸). However, none of the characterized single crystals contains a hydrogen-bond donor/acceptor in the aryl substituent at position 3 of the quinazolinone unit and information on inter­molecular inter­actions of such structures is still missing. The only example containing a carb­oxy­lic functionality at the 3-aryl substituent of quinazolin-4(3*H*)-one was analysed as a complex with *Staphylococcus aureus* at the PBP2a binding site (Bouley *et al.*, 2015 ▸).

## Synthesis and crystallization   

**Figure 5:**
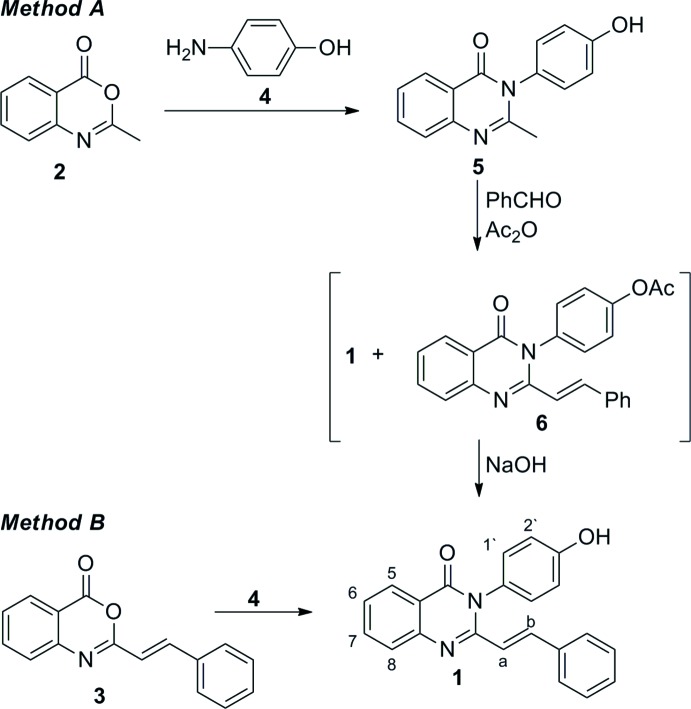
Synthesis of the title compound, **1**.

The title compound **1** was synthesized applying two pathways starting from 2-methyl (**2**) or 2-styryl (**3**) benzoxazin-4-one (methods *A* and *B*, respectively, Fig. 5 ▸).

***Method A***

2-Methyl benzoxazin-4-one (**2**) (0.263 g, 1.6 mmol) and 4-amino­phenol (**4**) (0.175 g, 1.6 mmol) in glacial acetic acid (2 ml) were refluxed for 7 h, then poured into crushed ice (50 ml) and filtered. Compound **5** was obtained as a greyish solid. Its spectroscopic data corresponded to those in the literature (Marinho & Proença, 2015 ▸). The crude product **5**, without further purification, was subjected to condensation with benzaldehyde analogously to a known method (Krastina *et al.*, 2014 ▸): 3-(4-hy­droxy­phen­yl)-2-methyl­quinazolin-4(3*H*)-one (**5**) (0.276 g, 1.1 mmol), benzaldehyde (0.27 g, 2.53 mol) and acetanhydride (0.5 ml) in acetic acid (4 ml) were refluxed for 8 h, poured into crushed ice (50 ml), filtered and air-dried. The mixture containing compounds **1** and **6** (0.25 g) was refluxed for 7 h in NaOH/methanol (5%, 5 ml), poured into crushed ice (50 ml), acidified with conc. hydro­chloric acid and filtered. The target compound **1** was obtained as a white solid with 53% (0.197 g) yield over two steps.

***Method B***

The title compound **1** was obtained as a by-product during the synthesis of 2-cinnamamido-*N*-(4-hy­droxy­phen­yl)benz­amide: benzoxazin-4-one **3** (1.00 g, 4 mmol) and 4-amino­phenol (**4**) (0.44 g, 4 mmol) were refluxed in toluene (5 ml) for 3 h, then the mixture was filtered. The title compound was isolated by crystallization from ethanol.

Single crystals suitable for X-ray analysis were obtained by slow evaporation from ethanol at room temperature (m.p. > 523 K).

Spectroscopic data: IR (KBr), ν, cm^−1^: 3300 (OH), 1655 (CON), 1150, 1515, 1470, 1450, 1340, 1225, 970, 775, 965. ^1^H NMR (300 MHz, DMSO-*d*_6_), δ (p.p.m.): 9.91 (1H, *s*, OH), 8.12 (1H, *d*, *J* = 7.8 Hz, H-5), 7.91–7.83 (2H, *m*, H-b, H-6/7), 7.76 (1H, *d*, *J* = 7.8 Hz, H-8), 7.52 (1H, *t*, *J* = 7.8 Hz, H-6/7), 7.41–7.33 (5H, *m*, Ph), 7.23 (2H, *d*, *J* = 8.6 Hz, H-1′), 6.94 (2H, *d*, *J* = 8.6 Hz, H-2′), 6.42 (1H, *d*, *J* = 15.4 Hz, H-a). ^13^C NMR (75 MHz, DMSO-*d*_6_), δ (p.p.m.): 161.5, 157.8, 152.0, 147.4, 138.6, 134.9, 134.7, 129.9, 129.8, 129.1, 127.9, 127.4, 127.1, 126.52, 126.47, 120.6, 120.2, 116.1. HRMS. Calculated [*M*+H]^+^, *m*/*z*: 341.1285. C_22_H_16_N_2_O_2_. Found, *m*/*z*: 341.1282.

## Refinement   

Crystal data, data collection and structure refinement details are summarized in Table 2 ▸. The C-bound H atoms were positioned geometrically and refined as riding on their parent atoms: C—H = 0.93 − 0.98 Å with *U*_iso_(H) = 1.5*U*_eq_(C) for methyl H atoms and 1.2*U*_eq_(C) for other H atoms. The H atom of the hydroxyl group was included in the position identified from a difference Fourier map and was then refined as riding: O—H = 0.82 Å with *U*_iso_(H) = 1.5*U*_eq_(O).

## Supplementary Material

Crystal structure: contains datablock(s) I. DOI: 10.1107/S2056989016004473/su5288sup1.cif

Structure factors: contains datablock(s) I. DOI: 10.1107/S2056989016004473/su5288Isup2.hkl

Supporting information file. DOI: 10.1107/S2056989016004473/su5288Isup3.cml

CCDC reference: 1468806

Additional supporting information:  crystallographic information; 3D view; checkCIF report

## Figures and Tables

**Table 1:** Hydrogen-bond geometry (Å, °) *Cg*3 and *Cg*4 are the centroids of the C12–C17 and C21–C26 rings, respectively.

*D*—H⋯*A*	*D*—H	H⋯*A*	*D*⋯*A*	*D*—H⋯*A*
O18—H18⋯O11^i^	0.82	1.84	2.654 (5)	172
C4—H4⋯*Cg*4^ii^	0.94	2.96	3.829 (5)	157
C16—H16⋯*Cg*3^i^	0.94	2.95	3.646 (5)	133

Symmetry codes: (i) 
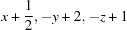
; (ii) 
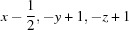
.

**Table 2:** Experimental details

Crystal data	
Chemical formula	C_22_H_16_N_2_O_2_
*M*_r_	340.37
Crystal system, space group	Orthorhombic, *P*2_1_ *n* *b*
Temperature (K)	173
*a*, *b*, *c* (Å)	5.3469 (2), 16.5139 (6), 19.8885 (10)
*V* (Å^3^)	1756.12 (13)
*Z*	4
Radiation type	Mo *K*α
μ (mm^−1^)	0.08
Crystal size (mm)	0.22 × 0.18 × 0.09

Data collection	
Diffractometer	Nonius KappaCCD
No. of measured, independent and observed [*I* > 2σ(*I*)] reflections	3862, 3862, 2236
(sin θ/λ)_max_ (Å^−1^)	0.649

Refinement	
*R*[*F* ^2^ > 2σ(*F* ^2^)], *wR*(*F* ^2^), *S*	0.068, 0.139, 1.03
No. of reflections	3862
No. of parameters	236
No. of restraints	1
H-atom treatment	H-atom parameters constrained
Δρ_max_, Δρ_min_ (e Å^−3^)	0.17, −0.19

Computer programs: *KappaCCD Server Software* (Nonius, 1997 ▸), *DENZO* and *SCALEPACK* (Otwinowski & Minor, 1997 ▸), *SIR2011* (Burla *et al.*, 2012 ▸), *ORTEP-3 for Windows* (Farrugia, 2012 ▸), *Mercury* (Macrae *et al.*, 2008 ▸), *SHELXL2015* (Sheldrick, 2015 ▸), *PLATON* (Spek, 2009 ▸) and *publCIF* (Westrip, 2010 ▸).

## supplementary crystallographic information

### Crystal data

**Table:**

C_22_H_16_N_2_O_2_	*D*_x_ = 1.287 Mg m^−^^3^
*M**_r_* = 340.37	Mo *K*α radiation, λ = 0.71073 Å
Orthorhombic, *P*2_1_*n**b*	Cell parameters from 6856 reflections
*a* = 5.3469 (2) Å	θ = 1.0–27.5°
*b* = 16.5139 (6) Å	µ = 0.08 mm^−^^1^
*c* = 19.8885 (10) Å	*T* = 173 K
*V* = 1756.12 (13) Å^3^	Plate, colorless
*Z* = 4	0.22 × 0.18 × 0.09 mm
*F*(000) = 712	

### Data collection

**Table:**

Nonius KappaCCD diffractometer	2236 reflections with *I* > 2σ(*I*)
Radiation source: fine-focus sealed tube	θ_max_ = 27.5°, θ_min_ = 3.2°
φ and ω scan	*h* = −6→6
3862 measured reflections	*k* = −21→21
3862 independent reflections	*l* = −25→25

### Refinement

**Table:**

Refinement on *F*^2^	Secondary atom site location: difference Fourier map
Least-squares matrix: full	Hydrogen site location: inferred from neighbouring sites
*R*[*F*^2^ > 2σ(*F*^2^)] = 0.068	H-atom parameters constrained
*wR*(*F*^2^) = 0.139	*w* = 1/[σ^2^(*F*_o_^2^) + (0.0478*P*)^2^ + 0.3939*P*] where *P* = (*F*_o_^2^ + 2*F*_c_^2^)/3
*S* = 1.03	(Δ/σ)_max_ < 0.001
3862 reflections	Δρ_max_ = 0.17 e Å^−^^3^
236 parameters	Δρ_min_ = −0.19 e Å^−^^3^
1 restraint	

### Special details

**Table:**

Geometry. All e.s.d.'s (except the e.s.d. in the dihedral angle between two l.s. planes) are estimated using the full covariance matrix. The cell e.s.d.'s are taken into account individually in the estimation of e.s.d.'s in distances, angles and torsion angles; correlations between e.s.d.'s in cell parameters are only used when they are defined by crystal symmetry. An approximate (isotropic) treatment of cell e.s.d.'s is used for estimating e.s.d.'s involving l.s. planes.

### Fractional atomic coordinates and isotropic or equivalent isotropic displacement parameters (Å^2^)

**Table:**

	*x*	*y*	*z*	*U*_iso_*/*U*_eq_	
N1	0.6956 (7)	0.7742 (2)	0.45545 (18)	0.0333 (9)	
C2	0.8449 (8)	0.7044 (3)	0.4584 (2)	0.0317 (10)	
N3	0.8479 (7)	0.6500 (2)	0.41192 (18)	0.0372 (9)	
C4	0.6912 (9)	0.6013 (3)	0.3061 (2)	0.0436 (12)	
H4	0.7956	0.5564	0.3096	0.052*	
C5	0.5358 (9)	0.6089 (3)	0.2518 (2)	0.0476 (13)	
H5	0.5349	0.5690	0.2188	0.057*	
C6	0.3791 (11)	0.6756 (3)	0.2457 (3)	0.0564 (15)	
H6	0.2738	0.6801	0.2086	0.068*	
C7	0.3795 (12)	0.7346 (3)	0.2939 (3)	0.0588 (15)	
H7	0.2750	0.7793	0.2895	0.071*	
C8	0.5362 (10)	0.7885 (3)	0.4018 (3)	0.0435 (12)	
C9	0.6946 (8)	0.6604 (3)	0.3564 (2)	0.0337 (11)	
C10	0.5376 (9)	0.7278 (3)	0.3501 (2)	0.0372 (11)	
O11	0.4054 (7)	0.8506 (2)	0.40120 (19)	0.0653 (12)	
C12	0.7042 (8)	0.8360 (3)	0.5076 (2)	0.0315 (10)	
C13	0.5181 (8)	0.8382 (3)	0.5557 (2)	0.0354 (11)	
H13	0.3953	0.7983	0.5563	0.042*	
C14	0.5143 (9)	0.8993 (3)	0.6027 (2)	0.0372 (11)	
H14	0.3905	0.9002	0.6356	0.045*	
C15	0.6941 (8)	0.9595 (3)	0.6013 (2)	0.0309 (10)	
C16	0.8821 (8)	0.9566 (3)	0.5533 (2)	0.0344 (11)	
H16	1.0059	0.9962	0.5528	0.041*	
C17	0.8859 (8)	0.8951 (3)	0.5064 (2)	0.0339 (11)	
H17	1.0110	0.8936	0.4739	0.041*	
O18	0.6763 (7)	1.01968 (18)	0.64797 (15)	0.0426 (8)	
H18	0.7583	1.0589	0.6356	0.064*	
C19	0.9961 (7)	0.6928 (3)	0.5188 (2)	0.0338 (11)	
H19	0.9602	0.7230	0.5570	0.041*	
C20	1.1845 (8)	0.6398 (3)	0.5204 (2)	0.0341 (10)	
H20	1.2220	0.6133	0.4804	0.041*	
C21	1.3390 (9)	0.6191 (3)	0.5794 (2)	0.0361 (11)	
C22	1.2883 (9)	0.6474 (3)	0.6437 (2)	0.0435 (12)	
H22	1.1527	0.6816	0.6508	0.052*	
C23	1.4371 (8)	0.6252 (3)	0.6973 (3)	0.0498 (15)	
H23	1.4006	0.6441	0.7402	0.060*	
C24	1.6404 (9)	0.5748 (3)	0.6875 (3)	0.0512 (14)	
H24	1.7421	0.5605	0.7235	0.061*	
C25	1.6909 (10)	0.5460 (3)	0.6243 (3)	0.0496 (13)	
H25	1.8265	0.5117	0.6176	0.060*	
C26	1.5421 (9)	0.5676 (3)	0.5706 (2)	0.0399 (12)	
H26	1.5779	0.5475	0.5280	0.048*	

### Atomic displacement parameters (Å^2^)

**Table:**

	*U*^11^	*U*^22^	*U*^33^	*U*^12^	*U*^13^	*U*^23^
N1	0.0351 (19)	0.030 (2)	0.035 (2)	0.0014 (17)	−0.0054 (19)	−0.0034 (17)
C2	0.031 (2)	0.027 (2)	0.037 (3)	0.003 (2)	−0.004 (2)	−0.002 (2)
N3	0.041 (2)	0.033 (2)	0.037 (2)	0.0064 (18)	−0.0066 (19)	−0.0067 (19)
C4	0.050 (3)	0.038 (3)	0.042 (3)	0.003 (2)	−0.001 (3)	−0.007 (2)
C5	0.052 (3)	0.051 (4)	0.040 (3)	−0.011 (3)	−0.001 (3)	−0.013 (3)
C6	0.066 (3)	0.065 (4)	0.038 (3)	−0.002 (3)	−0.017 (3)	−0.007 (3)
C7	0.067 (3)	0.059 (4)	0.050 (3)	0.012 (3)	−0.021 (3)	−0.003 (3)
C8	0.045 (3)	0.041 (3)	0.043 (3)	0.004 (3)	−0.012 (3)	−0.001 (2)
C9	0.036 (2)	0.034 (3)	0.031 (3)	−0.001 (2)	−0.004 (2)	−0.002 (2)
C10	0.044 (2)	0.038 (3)	0.030 (3)	0.004 (2)	−0.006 (2)	−0.001 (2)
O11	0.081 (3)	0.055 (3)	0.060 (3)	0.029 (2)	−0.030 (2)	−0.010 (2)
C12	0.031 (2)	0.031 (3)	0.032 (3)	0.003 (2)	−0.003 (2)	−0.003 (2)
C13	0.036 (2)	0.029 (3)	0.041 (3)	−0.009 (2)	−0.001 (2)	0.004 (2)
C14	0.039 (2)	0.037 (3)	0.036 (3)	−0.002 (2)	0.006 (2)	0.003 (2)
C15	0.040 (2)	0.027 (3)	0.026 (2)	−0.003 (2)	−0.003 (2)	0.001 (2)
C16	0.034 (2)	0.033 (3)	0.036 (3)	−0.007 (2)	0.001 (2)	0.000 (2)
C17	0.032 (2)	0.036 (3)	0.034 (3)	−0.001 (2)	0.004 (2)	0.001 (2)
O18	0.062 (2)	0.0355 (19)	0.0304 (17)	−0.0100 (17)	0.0041 (16)	−0.0058 (16)
C19	0.038 (2)	0.029 (3)	0.034 (3)	−0.002 (2)	−0.006 (2)	−0.002 (2)
C20	0.040 (2)	0.027 (2)	0.035 (3)	−0.002 (2)	−0.005 (2)	0.000 (2)
C21	0.037 (2)	0.030 (3)	0.041 (3)	−0.007 (2)	−0.012 (2)	0.001 (2)
C22	0.041 (3)	0.045 (3)	0.044 (3)	−0.003 (2)	−0.008 (2)	−0.004 (3)
C23	0.054 (3)	0.060 (4)	0.036 (3)	−0.010 (3)	−0.009 (2)	0.002 (3)
C24	0.047 (3)	0.060 (4)	0.047 (4)	−0.009 (3)	−0.018 (2)	0.011 (3)
C25	0.038 (3)	0.051 (3)	0.059 (4)	0.001 (2)	−0.007 (3)	0.016 (3)
C26	0.039 (2)	0.040 (3)	0.041 (3)	−0.001 (2)	−0.003 (2)	0.003 (2)

### Geometric parameters (Å, º)

**Table:**

N1—C8	1.385 (6)	C14—H14	0.9300
N1—C2	1.404 (5)	C15—O18	1.364 (5)
N1—C12	1.455 (5)	C15—C16	1.387 (6)
C2—N3	1.289 (5)	C16—C17	1.379 (6)
C2—C19	1.459 (6)	C16—H16	0.9300
N3—C9	1.385 (5)	C17—H17	0.9300
C4—C5	1.368 (7)	O18—H18	0.8200
C4—C9	1.398 (6)	C19—C20	1.335 (6)
C4—H4	0.9300	C19—H19	0.9300
C5—C6	1.390 (7)	C20—C21	1.475 (6)
C5—H5	0.9300	C20—H20	0.9300
C6—C7	1.367 (7)	C21—C22	1.389 (6)
C6—H6	0.9300	C21—C26	1.390 (6)
C7—C10	1.406 (7)	C22—C23	1.379 (6)
C7—H7	0.9300	C22—H22	0.9300
C8—O11	1.241 (6)	C23—C24	1.382 (7)
C8—C10	1.436 (6)	C23—H23	0.9300
C9—C10	1.400 (6)	C24—C25	1.370 (7)
C12—C17	1.378 (6)	C24—H24	0.9300
C12—C13	1.381 (6)	C25—C26	1.378 (7)
C13—C14	1.377 (6)	C25—H25	0.9300
C13—H13	0.9300	C26—H26	0.9300
C14—C15	1.383 (6)		
			
C8—N1—C2	121.5 (4)	C15—C14—H14	119.9
C8—N1—C12	116.7 (4)	O18—C15—C14	117.4 (4)
C2—N1—C12	121.8 (3)	O18—C15—C16	123.0 (4)
N3—C2—N1	123.3 (4)	C14—C15—C16	119.6 (4)
N3—C2—C19	119.4 (4)	C17—C16—C15	120.1 (4)
N1—C2—C19	117.3 (4)	C17—C16—H16	119.9
C2—N3—C9	118.6 (4)	C15—C16—H16	119.9
C5—C4—C9	120.6 (5)	C12—C17—C16	120.0 (4)
C5—C4—H4	119.7	C12—C17—H17	120.0
C9—C4—H4	119.7	C16—C17—H17	120.0
C4—C5—C6	120.6 (5)	C15—O18—H18	109.5
C4—C5—H5	119.7	C20—C19—C2	121.6 (4)
C6—C5—H5	119.7	C20—C19—H19	119.2
C7—C6—C5	120.2 (5)	C2—C19—H19	119.2
C7—C6—H6	119.9	C19—C20—C21	126.5 (4)
C5—C6—H6	119.9	C19—C20—H20	116.8
C6—C7—C10	120.1 (5)	C21—C20—H20	116.8
C6—C7—H7	119.9	C22—C21—C26	118.3 (4)
C10—C7—H7	119.9	C22—C21—C20	123.1 (4)
O11—C8—N1	119.7 (5)	C26—C21—C20	118.7 (4)
O11—C8—C10	124.9 (5)	C23—C22—C21	120.6 (5)
N1—C8—C10	115.5 (4)	C23—C22—H22	119.7
N3—C9—C4	119.4 (4)	C21—C22—H22	119.7
N3—C9—C10	121.7 (4)	C22—C23—C24	120.3 (5)
C4—C9—C10	118.9 (4)	C22—C23—H23	119.9
C9—C10—C7	119.7 (4)	C24—C23—H23	119.9
C9—C10—C8	119.5 (4)	C25—C24—C23	119.6 (5)
C7—C10—C8	120.7 (5)	C25—C24—H24	120.2
C17—C12—C13	120.1 (4)	C23—C24—H24	120.2
C17—C12—N1	120.4 (4)	C24—C25—C26	120.4 (5)
C13—C12—N1	119.3 (4)	C24—C25—H25	119.8
C14—C13—C12	120.1 (4)	C26—C25—H25	119.8
C14—C13—H13	120.0	C25—C26—C21	120.8 (5)
C12—C13—H13	120.0	C25—C26—H26	119.6
C13—C14—C15	120.1 (4)	C21—C26—H26	119.6
C13—C14—H14	119.9		
			
C8—N1—C2—N3	1.2 (7)	C8—N1—C12—C17	−95.4 (5)
C12—N1—C2—N3	−177.5 (4)	C2—N1—C12—C17	83.3 (5)
C8—N1—C2—C19	−176.7 (4)	C8—N1—C12—C13	80.1 (5)
C12—N1—C2—C19	4.7 (6)	C2—N1—C12—C13	−101.2 (5)
N1—C2—N3—C9	−0.6 (6)	C17—C12—C13—C14	−0.3 (6)
C19—C2—N3—C9	177.1 (4)	N1—C12—C13—C14	−175.8 (4)
C9—C4—C5—C6	0.2 (8)	C12—C13—C14—C15	1.1 (7)
C4—C5—C6—C7	0.2 (9)	C13—C14—C15—O18	178.2 (4)
C5—C6—C7—C10	−0.3 (9)	C13—C14—C15—C16	−1.8 (7)
C2—N1—C8—O11	179.4 (5)	O18—C15—C16—C17	−178.4 (4)
C12—N1—C8—O11	−1.9 (7)	C14—C15—C16—C17	1.6 (7)
C2—N1—C8—C10	−0.7 (6)	C13—C12—C17—C16	0.1 (6)
C12—N1—C8—C10	178.0 (4)	N1—C12—C17—C16	175.5 (4)
C2—N3—C9—C4	−178.7 (4)	C15—C16—C17—C12	−0.7 (7)
C2—N3—C9—C10	−0.3 (6)	N3—C2—C19—C20	17.8 (7)
C5—C4—C9—N3	178.0 (4)	N1—C2—C19—C20	−164.3 (4)
C5—C4—C9—C10	−0.5 (7)	C2—C19—C20—C21	−175.8 (4)
N3—C9—C10—C7	−178.0 (5)	C19—C20—C21—C22	6.9 (7)
C4—C9—C10—C7	0.4 (7)	C19—C20—C21—C26	−174.5 (4)
N3—C9—C10—C8	0.7 (7)	C26—C21—C22—C23	0.4 (7)
C4—C9—C10—C8	179.1 (4)	C20—C21—C22—C23	179.0 (4)
C6—C7—C10—C9	0.0 (8)	C21—C22—C23—C24	0.5 (7)
C6—C7—C10—C8	−178.7 (5)	C22—C23—C24—C25	−1.0 (7)
O11—C8—C10—C9	179.8 (5)	C23—C24—C25—C26	0.6 (8)
N1—C8—C10—C9	−0.2 (6)	C24—C25—C26—C21	0.3 (7)
O11—C8—C10—C7	−1.6 (8)	C22—C21—C26—C25	−0.8 (7)
N1—C8—C10—C7	178.5 (5)	C20—C21—C26—C25	−179.5 (4)

### Hydrogen-bond geometry (Å, º)

Cg3 and Cg4 are the centroids of the C12–C17 and C21–C26 rings, respectively.

**Table:**

*D*—H···*A*	*D*—H	H···*A*	*D*···*A*	*D*—H···*A*
O18—H18···O11^i^	0.82	1.84	2.654 (5)	172
C4—H4···*Cg*4^ii^	0.94	2.96	3.829 (5)	157
C16—H16···*Cg*3^i^	0.94	2.95	3.646 (5)	133

Symmetry codes: (i) *x*+1/2, −*y*+2, −*z*+1; (ii) *x*−1/2, −*y*+1, −*z*+1.
